# Prevalence, Awareness and Associated Risk Factors of Diabetes among Adults in Xi’an, China

**DOI:** 10.1038/s41598-017-10797-x

**Published:** 2017-09-05

**Authors:** Meiqin Hu, Yi Wan, Lifen Yu, Jing Yuan, Yonghong Ma, Bin Hou, Xun Jiang, Lei Shang

**Affiliations:** 1 Department of Health Statistics, School of Public Health, Fourth Military Medical University, Xi’an, 710032 Shaanxi China; 2 Xi’an Centre for Disease Control and Prevention, Xi’an, 710054 Shaanxi China; 3 Department of Health Services, School of Public Health, Fourth Military Medical University, Xi’an, 710032 Shaanxi China; 4 Department of Pediatrics, Tangdu Hospital, Fourth Military Medical University, Xi’an, 710038 Shaanxi China

## Abstract

The study aimed to investigate the prevalence, awareness, treatment and glycaemic control of diabetes and its associated risk factors among adults in Xi’an, China. We collected data among participants aged 18 years or older through a self-developed questionnaire and an additional health examination. A total of 8150 participants were included, with an overall prevalence of diabetes of 8.0%. Among 655 participants with diabetes, 52.5% were aware they had diabetes, 48.1% took antidiabetic treatment, and 19.1% had their fasting blood glucose level at less than 7.0 mmol/l. Older age, lower educational level, higher body mass index, larger waist circumference, having an unhealthy diet and having more comorbidities were positively associated with the risk of diabetes. Participants who were older, who had higher education and who had more comorbidities were more aware that they had diabetes. Being older age, having higher education and having more comorbidities were also factors for better treatment. Participants who were older were more likely to have their glucose level controlled. The prevalence of diabetes among adults in Xi’an is high, with suboptimal awareness, treatment, and glycaemic control rates. Comprehensive integrated strategies based on risk factors should be implemented to improve the prevention and glycaemic control of diabetes.

## Introduction

Diabetes is a major risk factor for morbidity and mortality worldwide, and it has become a significant public health problem in China^1–3^. High blood glucose accounts for 21% of all deaths from ischaemic heart disease and 13% of all deaths from stroke worldwide, with 84% of these cardiovascular deaths occurring in low- and middle-income countries^4^. It has been reported that Asia is the epicentre of the diabetes epidemic. People in Asia who develop diabetes tend to have lower body mass index (BMI), be younger, suffer longer from the complications of diabetes, and die sooner than people with diabetes in other regions^5, 6^. China, as the most populous country in Asia, has conducted several national surveys in recent decades that indicate that the prevalence of diabetes among adults has increased dramatically from 0.7% in 1980 to 2.5% in 1994, to 5.5% in 2000–2001 (2.7% in 2002), and to 9.7% in 2007–2008^1^ ^, 7–10^. In the most recent national survey conducted in 2010, the prevalence of diabetes was 11.6%, representing up to 113.9 million Chinese adults with diabetes^2^. Although the screening methods and diagnostic criteria were different in those national surveys, the results showed a staggering increase in diabetes among Chinese adults during the last three decades (Table 1). Reviews also confirmed this rising trend in the prevalence of diabetes^11, 12^.

**Table 1:** Summary of study procedure about nationwide diabetes epidemiological surveys in China.

No.	Study area	Study time	Sample Size	Age (years)	Screening Methods	OGTTconducted with the given condition	Diagnosis criteria	Sampling procedure	Prevalence (%)
1	Nationwide^7^	1980	304537	All age groups	Urine, 2 h blood glucose, OGTT	2 h plasma glucose ≥140 mg/dl	Self-defined	Not randomly, covering 14 provinces	0.7
2	Nationwide^8^	1994	213515	25–64	FCG OGTT	2hplasma glucose ≥6.67 mmol/l	WHO1985	Not randomly, covering 19 provinces	2.5
3	Nationwide^9^	2001	15,236	35–74	FPG	N/A	ADA 2002	First stage is not random, covering 10 provinces	5.5
4	Nationwide^39^	2002	51970	18–79	FPG, OGTT	FPG ≥ 5.5 mmol/l	WHO 1999	Multi-step random cluster, covering 31 provinces	2.6
5	Nationwide^10^	2002	47729	≥20	FPG,	N/A	ADA2009	Multi-step random cluster, covering 31 provinces	2.7
6	Nationwide^1^	2008	46239	≥20	FPG, OGTT	All except previous cases	WHO 1999	Two stages are not random, covering 14 provinces	9.7
7	Nationwide^2^	2010	98658	≥18	FPG, OGTT, HbA_1c_	All except previous cases	ADA 2010	Complex, multistage, probability sampling	11.6

Note: FPG = fasting plasma glucose level. FCG = fasting capillary whole blood glucose level. OGTT = oral glucose tolerance test; N/A: no available data; the diagnostic criteria of diabetes applied to this review were consistent except for study 1, 2 and 7. In study 2, diabetes was defined based on fasting capillary whole blood glucose level (fasting capillary whole blood glucose level ≥6.1 mmol/l) whereas other studies used plasma glucose measurement (FPG ≥ 7.0 mmol/l, 2 h PG ≥ 11.1 mmol/l during an OGTT, or previously diagnosed diabetes). In study 7, diabetes was defined based on previous plasma glucose measurement and glycosylated HbA_1c_ (HbA_1c_ ≥ 6.5%). Study 4 and 5 were from the same survey, the China National Nutrition and Health Survey, 2002.

Studies have investigated the prevalence of diabetes at both national and regional levels among Chinese adults^2, 13^. Some studies also focused on the awareness, treatment, and glycaemic control of diabetes, and reviews showed that there was no obvious improvement in diabetes awareness from 1979 to 2012^11, 13, 14^. It has been reported that maintaining blood glucose at a normal level could significantly lower the risk of diabetes-related complications, causing a delay in disease progression. However, studies about the associated risk factors of awareness, treatment and glycaemic control of diabetes are limited. Xi’an, which is the largest city in the northwest of China, is still an economically under- to intermediately developed area, where the staple food is made from wheat flour. The area has distinct geographic characteristics, cultural behaviours and lifestyles compared to the coastal, northeastern and southern regions of China, and all of these factors may affect the prevalence of diabetes. Furthermore, information on the status of diabetes among adults in Xi’an is scarce. The objective of this study is therefore to estimate the prevalence, awareness, treatment, and glycaemic control of diabetes based on fasting plasma glucose (FPG) and to explore the associated risk factors of diabetes among adults in Xi’an, China.

## Results

Of the 8401 participants aged 18 years or above who were recruited for the survey, participants who failed to attend the interview (*n* = 149) or did not complete the questionnaire (*n* = 24) were excluded. Additionally, 78 participants without a blood glucose measurement and who did not complete the health examination were excluded from the analysis. The remaining 8150 participants were included for analysis, with 4121 (50.6%) participants living in urban areas and 4051 (49.7%) of participants being male. The mean age of the participants for analysis was 41.0 ± 16.1 years old. The general characteristics of the participants are given in Table 2. The majority of participants had completed junior high school and were married/cohabiting. Most participants (50.4%) did manual work.

**Table 2:** General characteristics of participants (*n*, %).

Items	N (%)	Diabetes prevalence	Awareness^a^	Treatment^b^	Glycaemic control^c^
Total	8150	655(8.0)	344(52.5)	315(91.6)	125(39.7)
Age groups					
18–44 y	4959(60.8)	152(3.1)	39(25.7)	34(87.2)	12(35.3)
45–59 y	1907(23.4)	231(12.1)	125(54.1)	115(92.0)	45(39.1)
60y-	1284(15.8)	272(21.2)^§^	180(66.2)^§^	166(92.2)	68(41.0)
*P* values		<0.001	<0.001	0.414	0.539
Gender					
Males	4051(49.7)	321(7.9)	176(54.8)	157(89.2)	55(35.0)
Females	4099(50.3)	334(8.1)	168(50.3)	158(94.0)	70(44.3)
*P* values		0.709	0.246	0.106	0.092
Residential areas					
Urban	4121(50.6)	324(7.9)	195(60.2)	177(90.8)	74(41.8)
Rural	4029(49.4)	331(8.2)	149(45.0)^§^	138(92.6)	51(37.0)
*P* values		0.557	<0.001	0.541	0.383
Education					
≤Primary school	1176(14.4)	200(17.0)	115(57.5)	104(90.4)	41(39.4)
Junior school	3358(41.2)	255(7.6)	107(42.0)	99(92.5)	36(36.4)
Senior school or higher	3616(44.4)	200(5.5)^§^	122(61.0)	112(91.8)	48(42.9)
*P* values		<0.001	0.484	0.710	0.595
Marital status					
Single	1559(19.1)	28(1.8)	9(32.1)	9(100.0)	5(55.6)
Married/cohabiting	6294(77.2)	575(9.1)	297(51.7)	270(90.9)	105(38.9)
Separated/divorced/widowed	297(3.6)	52(17.5)^§^	38(73.1)^§^	36(94.7)	15(41.7)
*P* values		<0.001	0.001	0.475	0.583
Occupation					
Manual labors	4109(50.4)	341(8.3)	146(42.8)	131(89.7)	46(35.1)
Mental labors	1920(23.6)	102(5.3)	54(52.9)	50(92.6)	20(40.0)
Unemployed	2121(26.0)	212(10.0)^§^	144(67.9)^§^	134(93.1)	59(44.0)
*P values*		<0.001	<0.001	0.569	0.333
BMI					
Underweight	412(5.1)	10(2.4)	4(40.0)	4(100.0)	2(50.0)
Normal	4129(50.7)	210(5.1)	111(52.9)	99(89.2)	42(42.4)
Overweight	2652(32.5)	273(10.3)	141(51.6)	128(90.8)	51(39.8)
Obese	957(11.7)	162(16.9)^§^	88(54.3)	84(95.5)	30(35.7)
*P values*		<0.001	0.639	0.184	0.791
WC					
Normal	4145(50.9)	185(4.5)	92(49.7)	82(89.1)	35(42.7)
Central obese	4005(49.1)	470(11.7)^§^	252(53.6)	233(92.5)	90(38.6)
*P values*		<0.001	0.370	0.325	0.518
Comorbidity					
Hypertension & dyslipidaemia	328(4.0)	102(31.1)	82(80.4)	75(91.5)	30(40.0)
Hypertension only	1420(17.4)	233(16.4)	131(56.2)	121(92.4)	46(38.0)
Dyslipidaemia only	249(3.1)	42(16.9)	30(71.4)	27(90.0)	10(37.0)
None	6153(75.5)	278(4.5)^§^	101(36.3)^§^	92(91.1)	39(42.4)
*P values*		<0.001	< <0.001	0.818	0.918
Smoking					
Current smoker	2349(28.8)	168(7.2)	86(51.2)	72(83.7)	25(34.7)
Former smoker	225(2.8)	38(16.9)	22(57.9)	22(100.0)	7(31.8)
No smoking	5576(68.4)	449(8.1)^§^	236(52.6)	221(93.6)	93(42.1)
*P values*		<0.001	0.756	0.006	0.398
Alcohol drinking					
Yes	884(10.8)	66(7.5)	39(59.1)	35(89.7)	11(31.4)
No	7266(89.2)	589(8.1)	305(51.8)	280(91.8)	114(40.7)
*P values*		0.509	0.259	0.663	0.289
Diet					
Healthy diet	5270(64.7)	392(7.4)	213(54.3)	117(89.7)	50(42.7)
Unhealthy diet	2880(35.3)	263(9.1)^§^	131(49.8)	198(92.7)	75(38.1)
*P values*		0.007	0.255	0.237	0.414
Physical activity					
High	1845(22.6)	133(7.2)	69(51.9)	61(88.4)	23(37.7)
Moderate	2974(36.5)	235(7.9)	133(56.6)	125(94.0)	53(42.4)
Low	3331(40.9)	287(8.6)	142(49.5)	129(90.8)	49(38.0)
*P values*		0.069	0.421	0.767	0.879

^§^ *χ* ^2^ test *P* < 0.05; ^a^among all participants with diabetes; ^b^among those who were aware they had diabetes; ^c^among those who were treated; BMI: body mass index; WC: waist circumference.

### Prevalence, awareness, treatment and glycaemic control of diabetes

Among the 8150 participants who qualified for the study, 8.0% (*n* = 655) had diabetes, including self-reported previous diagnosed diabetes, and a FPG ≥ 7.0 mmol/L. There was no significant difference between males and females (7.9% in males and 8.1% in females, *P* = 0.709) or between participants in urban and rural areas (7.9% in urban and 8.2% in rural, *P* = 0.557). In the univariate analysis, older age, higher BMI, larger waist circumference (WC) and having more comorbidities were associated with an increased risk of diabetes, while a higher level of education was associated with a reduced risk of diabetes. Furthermore, a higher prevalence of diabetes was found among those who were separated/divorced/widowed, unemployed and former smokers than their counterparts (all *P* < 0.05) (Table 2). Participants with an older age, living in urban areas, being separated/divorced/widowed, and having more comorbidities were more aware of their diabetes. Only females were more likely to have their glucose level controlled.

Among the 655 participants with diabetes, 344 (52.5%) were aware they had diabetes; 315 (48.1%) took antidiabetic treatment, with 274 (41.8%) persons taking pharmacological and non- pharmacological treatment and the other 41 (6.3%) persons just receiving treatment through diet and exercise; and 125 (19.1%) had their blood glucose level at less than 7.0 mmol/l (Table 3). Still, there were 311 (47.5%) persons who did not know they had diabetes and 29 (4.4%) persons who knew they had diabetes but did not take any measures to control their blood glucose. Among those who were aware they had diabetes, the total treatment rate and pharmacological treatment rate were 91.6% and 79.7%, respectively. The optimal blood glucose level control rate was 39.7% among those who received the treatment and 36.1% among those who took pharmacological treatment. Compared to those living in rural areas, participants in urban areas were more aware they had diabetes and more likely to be taking treatment and controlling their blood glucose; however, this pattern was not found between men and women. There were no significant differences between those in urban areas and those in rural areas either for treatment among those who were aware of their diabetic conditions or in control among those who were treated, and similar patterns were found for men and women. In addition, a significant improvement in glycaemic control among those taking pharmacological treatment was observed.

**Table 3:** The rate of diabetes awareness, treatment, and glycaemic control among adults in Xi’an by residence areas and gender (*n*, %).

	Urban	Rural	*P* values	Males	Females	*P* values	Total
Awareness^&^(n = 655)	195(60.2)	149(45.0)	<0.001	176(54.8)	168(50.3)	0.246	344(52.5)
Treatment							
Among all persons with diabetes^&^(n = 655)	177(54.6)	138(41.7)	<0.001	157(48.9)	158(47.3)	0.246	315(48.1)
Among those who aware they had diabetes(n = 344)	177(90.8)	138(92.6)	0.541	157(89.2)	158(94.0)	0.106	315(91.6)
Pharmacological treatment							
Among all persons with diabetes^&^(n = 655)	155(47.8)	119(36.0)	0.002	139(43.3)	135(40.4)	0.455	274(41.8)
Among those who aware they had diabetes(n = 344)	155(79.5)	119(79.9)	0.931	139(79.0)	135(80.4)	0.751	274(79.7)
Glycaemic control							
Among all persons with diabetes^&^(n = 655)	74(22.8)	51(15.4)	0.016	55(17.1)	70(21.0)	0.213	125(19.1)
Among those taking treatment(n = 314)	74(41.8)	51(37.0)	0.383	55(35.0)	70(44.3)	0.092	125(39.7)
Among those taking pharmacological treatment^§^(n = 274)	61(39.4)	38(31.9)	0.205	40(28.8)	59(43.7)	0.010	99(36.1)

^&^Urban versus rural, *P* < 0.05; ^§^males versus females, *P* < 0.05.

### Risk factors associated with prevalence, awareness, treatment and glycaemic control of diabetes

The results from univariable and multivariable risk factor analysis are presented in Table 4. After controlling for other variables, older age, higher BMI, larger WC and having more comorbidities were independently, significantly, and positively associated with diabetes (hypertension & dyslipidaemia: OR = 3.68, 95% CI = 2.75–4.91; hypertension only: OR = 1.57, 95% CI = 1.27–1.95; dyslipidaemia only: OR = 2.39, 95% CI = 1.65–3.47). A higher educational level, having a healthy diet and being single were negatively associated with the prevalence of diabetes.

**Table 4:** Risk factors analyses on the prevalence, awareness, treatment and glycaemic control of diabetes ^a^(*OR*, 95% *CI*).

	Prevalence(n = 8150)		Awareness(n = 655)		Treatment(n = 655)		Glycaemic control(n = 655)	
	OR^b^, 95% CI	OR^c^, 95% CI	OR^b^, 95% CI	OR^c^, 95% CI	OR^b^, 95% CI	OR^c^, 95% CI	OR^b^, 95% CI	OR^c^, 95% CI
**Age (ref: 18–44, years)**								
45–59 y	4.41(3.56,5.46)	2.57(2.03, 3.26)	3.33(2.13,5.22)	2.83(1.73, 4.62)	3.35(2.11,5.32)	2.84(1.72, 4.67)	2.71(1.38,5.33)	2.56(1.30,5.06)
60y-	8.60(6.96,10.62)	4.07(3.13, 5.29)	5.43(3.48,8.46)	4.52(2.64, 7.74)	5.28(3.35,8.32)	4.37(2.55, 7.50)	3.79(1.97,7.26)	3.14(1.59,6.21)
**Education (ref: primary school)**								
Junior school	0.40(0.33,0.49)	0.73(0.58, 0.92)	0.54(0.37,0.79)	0.87(0.56, 1.33)	0.59(0.41,0.86)	0.95(0.62,1.45)	0.62(0.38,1.02)	—
Senior school or higher	0.28(0.23,0.35)	0.68(0.53, 0.86)	1.18(0.79,1.76)	1.80(1.07, 3.03)	1.22(0.82,1.81)	1.80(1.09,2.98)	1.22(0.76,1.95)	—
**Marital status (ref: married/cohabiting)**								
Single	0.19(0.13,0.27)	0.60(0.39, 0.90)	0.44(0.20,1.00)	—	0.53(0.24,1.20)	—	0.98(0.36,2.64)	—
Separated/divorced/widowed	2.16(1.58,2.95)	0.99(0.71, 1.39)	2.54(1.35,4.79)	—	2.53(1.37,4.66)	—	1.83(0.97,3.45)	—
**Occupation (ref: manual labors)**								
Manual labors	1.65(1.31,2.08)	—	0.64(0.41,1.01)	0.72(0.42, 1.24)	0.63(0.40,0.98)	0.70(0.41, 1.20)	0.64(0.36,1.16)	0.58(0.32,1.06)
Unemployed	2.02(1.57,2.58)	—	1.88(1.15,3.07)	1.42(0.79, 2.56)	1.82(1.12,2.96)	1.39(0.78, 2.47)	1.66(0.92,2.97)	1.24(0.66,2.30)
**BMI (ref: normal)**								
Underweight	0.47(0.25,0.90)	0.67(0.35, 1.30)	0.60(0.16,2.19)	—	0.74(0.20,2.71)	—	0.98(0.20,4.80)	—
Overweight	2.14(1.78,2.59)	1.35(1.09, 1.68)	0.95(0.66,1.37)	—	0.97(0.68,1.40)	—	0.87(0.55,1.38)	—
Obese	3.80(3.05,4.74)	2.01(1.54, 2.63)	1.10(0.73,1.66)	—	1.23(0.81,1.86)	—	0.91(0.54,1.53)	—
**WC (ref: normal)**								
Central obese	2.86(2.40,3.42)	1.34(1.07, 1.66)	1.23(0.87,1.73)	—	1.27(0.90,1.79)	—	1.03(0.66,1.60)	—
**Diet(ref: unhealthy diet)**								
Healthy diet	0.80(0.68,0.94)	0.83(0.70,0.99)	1.22(0.89,1.67)		1.27(0.93,1.75)		1.02(0.68,1.52)	
**Comorbidity (ref: none)**								
Hypertension and dyslipidaemia	9.56(7.34,12.45)	3.68(2.75, 4.91)	6.98(4.04,12.06)	5.45(3.06, 9.72)	5.45(3.28,9.05)	4.20(2.45, 7.18)	2.56(1.48,4.41)	—
Hypertension only	4.14(3.44,4.99)	1.57(1.27, 1.95)	2.18(1.53,3.12)	1.50(1.01, 2.23)	2.15(1.50,3.09)	1.50(1.01, 2.23)	1.44(0.90,2.31)	—
Dyslipidaemia only	4.32(3.04,6.15)	2.39(1.65, 3.47)	4.31(2.11,8.79)	3.83(1.77, 8.29)	3.58(1.82,7.06)	3.06(1.46, 6.39)	1.89(0.86,4.15)	—

CI: confidence interval; OR: odds ratio; BMI: body mass index; WC: waist circumference; ^a^among all participants with diabetes; ^b^the univariable estimates for each factor; ^c^the stepwise logistic regression was used for analysis with a 0.10 significance level for removal from the model and a significance level of 0.05 for addition to the model.

After adjustment, older age, higher educational level and having more comorbidities were associated with better awareness among all participants with diabetes (hypertension & dyslipidaemia: OR = 5.45, 95% CI = 3.06–9.72; hypertension only: OR = 1.50, 95% CI = 1.01–2.23; dyslipidaemia only: OR = 3.83, 95% CI = 1.77–8.29). Among all participants with diabetes, older age, higher educational level and having more comorbidities were associated with a higher rate of diabetic treatment (hypertension & dyslipidaemia: OR = 4.20, 95% CI = 2.45–7.18; hypertension only: OR = 1.50, 95% CI = 1.01–2.23; dyslipidaemia only: OR = 3.06, 95% CI = 1.46–6.39). Participants who were older were more likely to control their glucose level (45–59 y: OR = 2.56, 95% CI = 1.30–5.06; 60 y and above: OR = 3.14, 95% CI = 1.59–6.21).

## Discussion

This population-based study showed that the prevalence of diabetes among adults in Xi’an was 8.0%, and that among all participants with diabetes, 52.5% were aware they had diabetes, 48.1% took antidiabetic medication, and 19.1% had their fasting glucose level controlled within 7.0 mmol/l. Diabetes was positively associated with older age, lower educational level, being married/cohabiting, higher BMI, larger WC, having unhealthy diet and having more comorbidities. Among all participants with diabetes, those who were older, with higher educational level and who had more comorbidities were more aware they had diabetes and received a higher rate of diabetic treatment. Furthermore, older participants were more likely to control their glucose level.

The prevalence of diabetes was surprisingly high among adults in Xi’an, with approximately 1 in 12 adults aged 18 years or above suffering from diabetes. Taking into account diabetes-related complications, including both microvascular disorders (e.g., neuropathy, nephropathy and retinopathy) and macrovascular disorders (e.g., cardiovascular disease, stroke and peripheral vascular disease) as well as diabetes-associated cancers^15^, diabetes imposes heavy health care costs on the health care system and becomes a huge financial burden on patients, their families and society as a whole^16, 17^. However, the prevalence of diabetes found in our study was much lower than the findings reported in the national surveys conducted in both China (11.6%)^2^ and the USA (12–14% in 2011–2012)^18^. Aside from the large geographical variation, the reason for the major difference in the prevalence of diabetes could be attributed to the different diagnostic criteria used. Specifically, our research defined diabetes based on FPG (≥126 mg/dL) alone, whereas the 2010 American Diabetes Association criteria for the diagnosis of diabetes that was used in Xu’s and Menke’s surveys included a glycosylated haemoglobin (HbA_1c_) level of 6.5% or greater, an FPG level of 126 mg/dL or greater, and a 2-hour plasma glucose (2 h PG) level of 200 mg/dL or greater. This was confirmed by Menke’s survey, in which the prevalence of diabetes based on HbA_1c_, FPG and 2 h PG (14.3%) was higher than that based only on HbA_1c_ and FPG (12.3%)^18^. In addition, with FPG alone, the prevalence of diabetes in our study was equal to that in Xu’s study, which found 4.5% for newly detected diabetes and 3.5% for previously diagnosed diabetes^2^. Our result was also lower than that of a survey done in Beijing (11.0%)^13^, but higher than that from a study done in a rural area in Jilin province (7.2%)^19^ and in two counties in Jiangsu province (7.31%)^20^. The different levels of economic development and urbanization, which were associated with a nutritional transition and changes in lifestyle, could be part of the reason for this discrepancy. This finding was supported by a review^12^ that reported that region-pooled prevalence of diabetes in the most developed eastern region in China was higher than that in the western region with regional levels of economic development. Additionally, geographic visualization at the provincial level indicated that there was a clear negative gradient in diabetes prevalence from urban areas with high socioeconomic conditions to rural areas with low socioeconomic conditions.

The common risk factors of age and obesity, indicated by either BMI or WC, were found to be positively associated with the prevalence of diabetes in our study. This result was consistent with the reviews of previous epidemiological studies^12, 21^. Considering that the elderly population, i.e., people aged 65 years or older, in Xi’an has increased to 8.46% in 2010^22^ and that the prevalence of obesity among adults has increased to 11.7% in the present survey, public health programmes need to pay urgent attention to the ageing population and to those who are obese. No significant differences were found in this study with regard to sex. This finding was in accordance with a previous review that concluded that there was an inconsistent association between sex and diabetes^21^. In contrast to other studies^12, 21^, we did not find a higher prevalence of diabetes among urban residents compared with rural residents. The rapid expansion of urbanization and the changing lifestyles of some residents in rural areas could explain the smaller gap between urban and rural areas in Xi’an. A systematic review with meta-analyses demonstrated that the Mediterranean diet was associated with better glycaemic control and suggested that it was suitable for the overall management of type 2 diabetes^23^. Compared with the Mediterranean diet, the diet for residents in Xi’an is unhealthy and includes highly refined flour-based food along with insufficient vegetables and fruits. In this study, unhealthy diet associated with the prevalence of diabetes was found, which was in accordance with previous findings. Both hypertension and dyslipidaemia have been found to be associated with diabetes in previous studies^19, 24^. This association was also found to be the case in our study. Furthermore, a higher risk of diabetes was found among participants who suffered from both hypertension and dyslipidaemia. One plausible explanation for this finding could be that patients who suffer from more diseases could be more susceptible to the development of diabetes. Educational level, one indicator of socioeconomic status, was negatively associated with the prevalence of diabetes in Xi’an. Similar findings were observed in other surveys^25, 26^. Furthermore, a meta-analysis concluded that the risk of getting type 2 diabetes was associated with low socioeconomic status based on education, occupation and income in high-income countries, whereas the strength of the association was less consistent in low- and middle-income countries^27^. Education may have an effect on the prevalence of diabetes, as it improves one’s ability to utilize relevant health information. In addition, being single was found to be associated with a reduced risk of diabetes in our findings. This finding was consistent with that of the study from the rural areas of Jilin province^19^. Compared with married/cohabiting participants, separated/divorced/widowed participants had a higher risk of diabetes even after adjusting for other factors. However, this finding was not significant in our study.

In this study, the awareness, treatment and glycaemic control among participants with diabetes was 52.5%, 48.1% and 19.1%, respectively, and it was expected to be 52.5%, 41.8% and 15.1% based on the definitions used in previous studies. Our results were higher than those found in 2010 (30.1%, 25.8%, and 10.2%, respectively)^2^ and those found in other studies in China (Shanghai^24^: 28.06%, 25.85%, and 12.42%; rural area in Shandong province^28^: 34.8%, 30.6%, and 11.5%) but were lower than those in Switzerland (65.3%, 56.4% and 18.9%, respectively)^29^ and those from some other studies in China (Jilin province^14^: 64.1%, 52.9% and 23.3%; Jiangsu province^20^: 58.35%, 51.87% and 14.12%; Zhejiang province^30^: 59.19%, 46.63% and 23.87%; Rural Diab study^26^: 60.11%, 54.85%, 18.77%). One plausible explanation for these differences could be the later survey time during which more campaigns against diabetes were launched. Another important potential factor was that all community health clinics or health centres in Xi’an, covering both urban and rural areas, had been involved in the management of diabetes after the release and implementation of Chinese guidelines for the management of diabetes.

Consistent with the findings from Zhejiang province^30^, age was positively associated with the awareness of diabetes. This result could be because people accumulate more knowledge about diabetes as their age increases and because the course of diabetes is relatively long. Diabetic patients with hypertension and dyslipidaemia were more likely to suffer from complications and therefore were more likely to contact health professionals and seek medical treatment. Thus, the rates of awareness and treatment were much higher among those patients. Additionally, we found diabetic patients with dyslipidaemia had higher rates of awareness and treatment than those with hypertension. The reason for this result needs further research. Education and occupation were marginally and positively associated with the awareness and treatment of diabetes. This result was also found in studies on the relationships of socioeconomic status (indicators: education, occupation and income) and diabetes^20, 25^.

Several limitations of this study deserve to be mentioned. Firstly, considering the limited study time during which the voluntary participants were available, the cost of this large population-based study, and the poor testing conditions in local clinics and health care centres, only FPG, without other glycaemic indexes -2 h PG or HbA_1c_, was used for the diagnosis of diabetes. Hence, the prevalence of diabetes in our study was underestimated. However, all participants with known diabetes were confirmed through their medical records. Secondly, one important risk factor, diabetes family history, was not included in this study, which might also be associated with low rate of awareness, treatment and glycaemic control. Previous research has shown that awareness and treatment of diabetes were higher in participants with a family history of diabetes^14^. Thirdly, distinguishing the type of diabetes is challenging, as it requires relatively sophisticated laboratory tests for pancreatic function. With limited resources, the type of diabetes was not distinguished in this study. Nevertheless, according to World Health Organization (WHO) reports, type 2 diabetes is the predominant form of diabetes in adults, making up approximately 85–90% of all cases of diabetes. In the study, 6 in 10 diabetic patients who were underweight according to their BMI did not know they had diabetes, hence, it is highly likely that they had type 1 diabetes. Considering that there are approximately 6,000 underweight diabetic patients who are unaware that they have diabetes among the entire Xi’an population of 8 million, these highly likely type 1 diabetes patients may be delayed in receiving lifesaving insulin treatment. Finally, due to the cross-sectional nature of our study and the potential reverse causation bias, the associations between some risk factors and the prevalence, awareness, treatment and glycaemic control of diabetes were unexpected. Despite these limitations, this study presented representative data on the prevalence, awareness, treatment and glycaemic control of diabetes among adults in Xi’an, which were targeted to a large population of eight million residents in the northwest of China. It also provided evidence for further management and control of diabetes at a population level.

In conclusion, the prevalence of diabetes was high in Xi’an, with suboptimal awareness, treatment, and glycaemic control rates. The prevalence was slightly higher than that in intermediately developed regions in China, therefore, we could deduce that it is higher in the northwest of China, where economic development has been relatively slow and the residents have had a similar lifestyle to those in Xi’an, with a diet consisting predominantly of flour-based foods and insufficient vegetables. The unprecedented high prevalence of diabetes among adults highlights the urgent need for more aggressive public health strategies and improved screening of the target population: older people, people with a lower educational level, obese/central obese people, people with an unhealthy diet and people with hypertension and dyslipidaemia.

## Methods

### Study design and participants

The study was part of a cross-sectional, population-based epidemiology survey conducted from October to December 2013. The design and methods of the survey have been described previously^31^. A stratified multiple-stage sampling method was used to select a representative, noninstitutionalized sample of adults from all 14 districts (urban) and counties (rural) in Xi’an. Following the sequence of community/town-residential area/village, a total of 8401 residents aged 18 years and above were sampled from selected residential areas/villages and stratified by age and sex according to the data of the Sixth Population Census in Xi’an, 2010. Participants who were aged ≥18 years, who had lived for more than six months at their current residence, and who were willing to participate were included in the survey. Individuals with dementia, schizophrenia, or serious illnesses, and those who were pregnant or who were unable to communicate effectively were excluded from the sample.

The study was approved by the Ethical Review Committee of Fourth Military Medical University and the Xi’an Centre for Disease Control and Prevention and was conducted in accordance with the Declaration of Helsinki. Written informed consent was obtained from each participant prior to the survey.

### Data collection

The participants were recruited to the community clinics or health care centres in their residential areas. The health examinations and questionnaire interviews were administered on-site by trained investigators using a standard protocol. The health examination involved the measurement of height, weight, WC, blood pressure (BP) and FPG. The questionnaire interview included information on socio-demographic and lifestyle factors (such as smoking, alcohol drinking, diet and physical activity). Self-reported histories of diabetes, hypertension and dyslipidaemia were recorded for each participant.

Anthropometric measurements of height, weight and WC were measured according to the standard protocol^32^. BMI was calculated as weight in kilograms divided by height in metres squared. Systolic and diastolic BP on the right arm were measured three consecutive times with standardized mercury sphygmomanometers according to the 1999 World Health Organization/International Society of Hypertension guidelines^33^. The mean of the latter two measurements was used for analysis. Venous blood samples were collected from all participants after an overnight fast using vacuum blood collection tubes containing anticoagulant sodium fluoride and were centrifuged on-site within 2 hours. FPG was measured in the laboratories of local hospitals within 24 hours using glucose oxidase or hexokinase methods.

### Quality control

Strict protocols were implemented to ensure the validity and reliability of the data. All investigators were uniformly trained and certified to conduct the face to face questionnaire interviews and to use the measurement instruments. Repeated interviews or examinations were conducted if missing information or logic errors were found. All measuring instruments and laboratories were standardized before the survey. All data were doubly entered into the database established by Epidata 3.1 software (Epidata Association, Odense, Denmark) and then compared and corrected for errors.

### Definitions

Diabetes was defined as a FPG level ≥7.0 mmol/L, and/or a self-reported previous diagnosis of diabetes (other than pregnancy) by a healthcare professional. Awareness of diabetes was defined as a self-reported history of any previous healthcare professional’s diagnosis of diabetes (other than pregnancy) before the survey. Treatment of diabetes was defined as the self-reported taking of insulin or oral hypoglycemic agents and/or taking non-pharmacological therapy (including diet and exercise) of those who reported a diagnosis of diabetes. Glycaemic control of diabetes was defined as the treatment of diabetes associated with an average FPG < 7.0 mmol/L.

Hypertension was defined as an average BP ≥ 140/90 mmHg, and/or a self-report of undertaking treatment with antihypertensive medication in the last two weeks^34^. Dyslipidaemia was defined as a self-reported history of previously diagnosed dyslipidaemia by a health professional before the survey. Smoking was defined as having smoked 100 cigarettes in one’s lifetime and/or currently smoking. Alcohol drinking was defined as having one drink at least once per week during the previous 12 months. Dietary assessment was through the Chinese food frequency questionnaire including all major food groups in the previous year (i.e., cereals, meats, fruits, vegetables, poultry, dairy products, beverages), and was grouped into healthy and unhealthy diet based on the WHO recommendations^35^. Physical activity, assessed by the global physical activity questionnaire, was divided into low, moderate and high according to the criteria used in other studies^36, 37^. According to criterion for Chinese populations, body size was categorized into underweight (BMI < 18.5 kg/m^2^), normal (BMI ≥ 18.5 kg/m^2^ and BMI < 24 kg/m^2^), overweight (BMI ≥ 24 kg/m^2^ and BMI < 28 kg/m^2^), and obese (BMI ≥ 28 kg/m^2^)^38^. WC was classed into normal (<85 cm for males and <80 cm for females) and central obese (≥85 cm for males and ≥80 cm for females)^38^.

### Statistical methods

Descriptive statistics were calculated and presented as the mean (standard deviation) for continuous variables and the frequency (percentage) for categorical variables. Pearson’s chi-square test was used for categorical variables, and the Cochran–Mantel–Haenszel test was used for ranked variables. Multivariate stepwise logistic regression was employed to explore the associated risk factors and expressed in odds ratios (OR) and 95% confidence intervals (95% CI). For the analyses, awareness, treatment and glycaemic control were strictly restricted to participants with diabetes. All statistical analyses were conducted using the Statistical Analysis System software package, version 9.2 (SAS Institute, Cary, NC, USA). All hypothesis tests were two-sided, and *P* < 0.05 was considered as statistically significant.
